# Relation-Based Deep Attention Network with Hybrid Memory for One-Shot Person Re-Identification

**DOI:** 10.3390/s21155113

**Published:** 2021-07-28

**Authors:** Runxuan Si, Jing Zhao, Yuhua Tang, Shaowu Yang

**Affiliations:** State Key Laboratory of High Performance Computing, College of Computer, National University of Defense Technology, Changsha 410000, China; sirunxuan14@nudt.edu.cn (R.S.); zhaojing@nudt.edu.cn (J.Z.); yhtang@nudt.edu.cn (Y.T.)

**Keywords:** hybrid memory, attention, Re-identification, one shot

## Abstract

One-shot person Re-identification, which owns one labeled sample among numerous unlabeled data for each identity, is proposed to tackle the problem of the shortage of labeled data. Considering the scenarios without sufficient labeled data, it is very challenging to keep abreast of the performance of the supervised task in which sufficient labeled samples are available. In this paper, we propose a relation-based attention network with hybrid memory, which can make full use of the global information to pay attention to the identity features for model training with the relation-based attention network. Importantly, our specially designed network architecture effectively reduces the interference of environmental noise. Moreover, we propose a hybrid memory to train the one-shot data and unlabeled data in a unified framework, which notably contributes to the performance of person Re-identification. In particular, our designed one-shot feature update mode effectively alleviates the problem of overfitting, which is caused by the lack of supervised information during the training process. Compared with state-of-the-art unsupervised and one-shot algorithms for person Re-identification, our method achieves considerable improvements of 6.7%, 4.6%, and 11.5% on Market-1501, DukeMTMC-reID, and MSMT17 datasets, respectively, and becomes the new state-of-the-art method for one-shot person Re-identification.

## 1. Introduction

With the development of smart vision in the field of public safety and video surveillance, person reidentification (Re-ID) [1] gradually has become an attractive research focus. The main task of Re-ID is to retrieve the same identity for a query person among different cameras and views. With the introduction of deep neural networks such as CNN, some supervised methods [2] for person Re-ID have made impressive progress. However, considering that it is labor intensive to annotate all kinds of datasets, many researchers have begun to study semi-supervised [3,4] and unsupervised [5,6] learning methods for person Re-ID to adapt better to real-world applications.

Recently, the research on person Re-ID pays more attention to unsupervised domains and attention mechanisms. The unsupervised methods can be divided into two aspects. One is the purely unsupervised learning (USL) method, which gradually exploits pseudo labels from the dataset by clustering strategy and similarity metric without any supervised information [7,8,9]. Cluster Contrast [10] represents the state-of-the-art performance in this type of method. The other is the unsupervised domain adaptation (UDA) person Re-ID [11,12,13], which fine-tunes the model on the unlabeled target dataset after pretraining model on a labeled source dataset. In this way, more information can be used for learning. Therefore, the performance of UDA is usually superior to USL due to the external source domain knowledge. However, the generalization of this method is relatively weak, especially when it comes to a situation where no labeled source dataset can be accessed; in these cases, UDA will not be applicable. In fact, completely unsupervised methods are too extreme. Specifically, in USL methods, it may lead to inaccurate cluster centers without any supervised information, which will greatly affect the performance of the model. In UDA methods, the dependence on the source domain limits the generalization ability of the model. Therefore, this study adopts a semi-supervised form to avoid the two problems above on the one hand, and on the other hand, it is feasible to annotate a small number of data in the semi-supervised case. In recent years, few-shot learning, which makes full use of the small labeled data for machine learning has been extensively studied. Our work is specifically aimed at the case in which only a small number of data need to be labeled with low cost, and a significant improvement can be achieved for the Re-ID task. Our method can also be extended to other types of Re-identification tasks.

As for the one-shot learning, it means that the dataset owns one labeled sample for each identity among numerous unlabeled data for the task of deep learning. As a video-based one-shot method, EUG [14] proposes a progressive sampling strategy to make full use of one-labeled tracklet of each identity. PPLS [15] makes progress for the image-based Re-ID task through introducing the data augmentation technology. However, considering the hysteresis of their algorithms of exploiting pseudo labels, the research of one-shot Re-ID has more room for development. To extract discriminative features for model training, many researchers [16,17] focus on the studies of the attention mechanism. Relation-aware global attention (RGA) [18] proposes a relation-aware global attention module that performs well in the supervised person Re-ID task.

**Figure 1:**
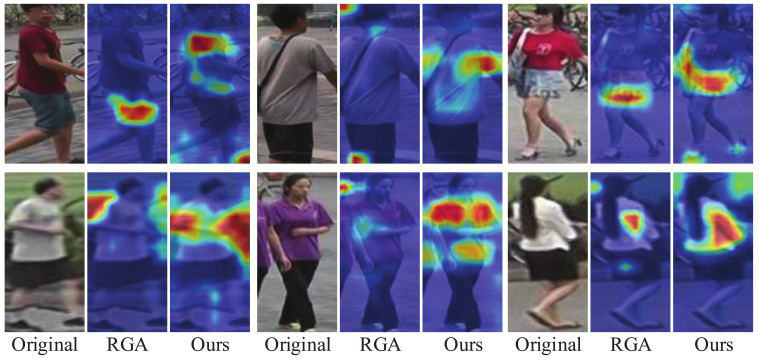
Examples of images that highlight important feature areas obtained from RGA and our method. For the color intensity, red indicates a large value while blue indicates a small one. Our work can capture wider and more accurate person identity information, compared with the RGA method.

Inspired by the above methods, we proposed a relation-based attention deep network with hybrid memory for one-shot person Re-ID in this work. Specifically, we first designed a hybrid memory that dynamically stores and updates the one-shot data features and unlabeled cluster features to train the one-shot data and unlabeled data jointly, which makes full use of the labeled information to improve the Re-ID performance. At the same time, to overcome the overfitting problem caused by the scarcity of one-shot data, we proposed a fine-tuning update mechanism that contributes to the Re-ID task. Considering that the easy examples with large instance numbers will overwhelm training and lead to degenerate models, we chose the hard instance [19] feature to update our hybrid memory. In addition, to transfer the attention mechanism to our scenario where labeled data is scarce, we designed a relation-based attention module that can address the global features and suppress interference from the environment. Taking into account the rapid development of unsupervised algorithms, we adopted the most representative clustering method to study our one-shot person Re-ID task. We evaluated the proposed method on three public image-based person Re-ID datasets, and the experiments demonstrate that our relation-based attention method yields better performance, compared with the state-of-the-art Re-ID approaches. Specifically, it achieves considerable improvements of 6.7%, 4.6%, and 11.5%, compared to the state-of-the-art unsupervised methods on the Market-1501, DukeMTMC-reID, and MSMT17 datasets, respectively. As for the state-of-the-art one-shot method, our work outperforms it by up to 43.7% and 35.2% on Market-1501 and DukeMTMC-reID, respectively, which mainly attributes to the introduction of the clustering algorithm. Intuitively, in Figure 1, we compared our relation-based attention method with RGA. It is evident that in terms of the effect of the attention mechanism, our method is significantly better than RGA.

The contributions of our work can be summarized as follows:

1. We designed a hybrid memory to train the one-shot data and unlabeled data jointly that makes full use of the labeled information to improve the Re-ID performance;
2. A fine-tuning update mechanism for our hybrid memory was proposed to overcome the overfitting problem caused by the scarcity of one-shot data for the Re-ID task;
3. Especially for the scenario where labeled data are scarce, we built a relation-based attention module that can address the global features while suppressing interference from the environment.

## 2. Related Works

### 2.1. Attention Models

By focusing on important features and suppressing irrelevant areas, the attention mechanism is well suitable for the person Re-ID task by extracting key features for person images where there is background clutter, occlusion, etc. A common research direction is to learn the attention mechanism through convolutional functions with small receptive fields on feature maps. Mixed high-order attention network (MHN) [20] is proposed to utilize the complex and high-order statistics information in the attention mechanism to capture the subtle differences among pedestrians. Harmonious attention CNN (HA-CNN) [21] learns soft pixel attention and hard regional attention, along with simultaneous optimization of feature representations jointly. Mancs [22] makes full use of the attention mechanism to solve the problem of character dislocation and appropriately sample the ranking loss to obtain a more stable character representation. However, the above methods only focus on the network convolutional functions and thus ignore the contextual information among the Re-ID task. Therefore, it is intuitively believed that the study of adding contextual information to the attention mechanism will make further progress.

To integrate more contextual information, convolutional block attention module (CBAM) [16] uses a 7 × 7 size filter over the spatial features to produce a spatial feature map. None-local [23] takes local and non-local attention blocks to extract features that capture the long-range dependencies between pixels and pays more attention to the challenging parts. RGA [18] explores the respective global scope relations for each feature node to learn attention with pairwise correlations. However, all these approaches are designed for supervised person Re-ID. Considering the more challenging task without enough labeled data, we need to design a special attention module for the one-shot person Re-ID.

### 2.2. Unsupervised Person Re-ID

Unsupervised person Re-ID can be summarized into two categories. The first type is purely unsupervised learning (USL) person Re-ID, which trains the model only with unlabeled data. The second is unsupervised domain adaptation (UDA), which integrates the transfer learning method to improve the performance of Re-ID. For USL person Re-ID, PAST [24] takes the conservative stage to capture the local structure of target-domain data points and optimizes the network by appending a changeable classification layer to the last layer of the model at the promoting stage. BUC [8] regards each individual sample as a different identity and gradually groups similar samples into one identity for clustering. MMCL [9] works with a memory-based nonparametric classifier and integrates multilabel classification and single-label classification jointly. Cluster contrast [10] builds a cluster-level memory dictionary and updates the cluster feature vectors in a consistent manner. As for the UDA person Re-ID, Yu et al. [11] propose a soft multilabel-guided hard negative mining method to learn a discriminative embedding for the unlabeled target domain. Hetero-homogeneous learning (HHL) [12] introduces the camera invariance and domain connectivity to improve the unsupervised training process. Furthermore, Zhong et al. [13] propose a new framework including exemplar invariance, camera invariance, and neighborhood invariance, compared to their previous work, HHL. AD cluster [25] incorporates style-translated images generated by GAN to enrich the diversity of instance features. SPCL [7] designs a novel self-paced contrastive learning framework that generates more reliable clusters to fine-tune the hybrid memory containing both source and target domain features gradually. In this paper, we focus on the limited labeled data from the dataset itself when no large amounts of labeled data from different domains can be obtained to improve the performance of person Re-ID.

### 2.3. Few-Shot Person Re-ID

Few-shot learning usually refers to the model learning on a data set with a small number of labeled data, and one-shot learning is a special case of few-shot tasks. One-shot learning can be divided into video-based and image-based tasks. For the video-based task, EUG [14] takes a progressive sampling strategy to make full use of one-labeled video tracklet to improve the accuracy of the pseudo labels annotating. For the image-based person Re-ID task, Wu et al. [26] propose a gradual sampling strategy to gradually increase the number of selected pseudo-labeled samples. PPLS [15] iteratively annotates pseudo labels for unlabeled data and takes a new sampling mechanism, which aims to label pseudo-labeled samples with unlabeled data based on the distance matrix. However, the above methods all obtain pseudo labels based on the identity category, which highly depends on the one-shot labeled data. This way will inevitably introduce many errors. In this paper, for the first time, a clustering algorithm is introduced into the one-shot task, using one-shot labeled data to fine-tune the deep network to improve the performance of the person Re-ID task.

## 3. Proposed Method

For one-shot person Re-ID, we propose a relation-based module that can extract important features and a hybrid memory that introduces one-shot samples to improve unsupervised training efficiently.

In this section, we first briefly look back at cluster contrast [10] in Section 3.1, which is an unsupervised method for person Re-ID based on SPCL [7]. Then, we introduce the hybrid memory for one-shot person Re-ID in Section 3.2. Finally, we describe our well-designed relation-based attention module in Section 3.3.

### 3.1. Cluster Contrast Revisit

On the one hand, cluster contrast, such as SPCL, chooses a clustering algorithm, such as DBSCAN [27] to generate pseudo labels, and a designed contrastive function is used to compute the loss between the query instances and the memory dictionary in the end. On the other hand, the memory mechanism of cluster contrast is different from SPCL. Each cluster is represented as a single feature vector, and the cluster feature vector is updated using the batch hard query instance feature which represents the most dissimilar query instance to cluster feature inside one mini-batch. Cluster contrast can be divided into three stages including memory initialization, memory update, and neural network training.

At the memory initialization stage, the feature of a random instance in the cluster which comes from the clustering algorithm is used to initialize the cluster feature. Similar to batch hard triplet loss [19], the hardest query instance inside one batch is selected to update the cluster feature. For a certain cluster with person identity *i*, its feature vector is updated as follows:

(1) $$\begin{array}{cc} q_{\mathrm{hard}\;} & \leftarrow \underset{q}{\arg\min}q\cdot c_{i},q\in Q^{i}\\ c_{i} & \leftarrow m\cdot c_{i}+\left(1-m\right)\cdot q_{\mathrm{hard}\;}, \end{array}$$

where the batch hard instance $q_{\mathrm{hard}\;}$ is the instance with the minimum similarity to the cluster feature $c_{i}$. $Q^{i}$ is the instance features set with cluster *i* in the current batch. *m* is the momentum update hyperparameter. During the training stage, InfoNCE [28] loss is used as follows:

(2) $$L_{q}=-\log\frac{\exp\left(q\cdot c^{+}\right)/\tau }{\sum_{i=0}^{K}\exp\left(q,c_{i}\right)/\tau }$$

where $c^{+}$ is the positive cluster feature vector to query instance *q* and τ is a temperature hyperparameter. More details about cluster contrast can be accessed in [10].

### 3.2. Hybrid Memory for One-Shot Learning

**Figure 2:**
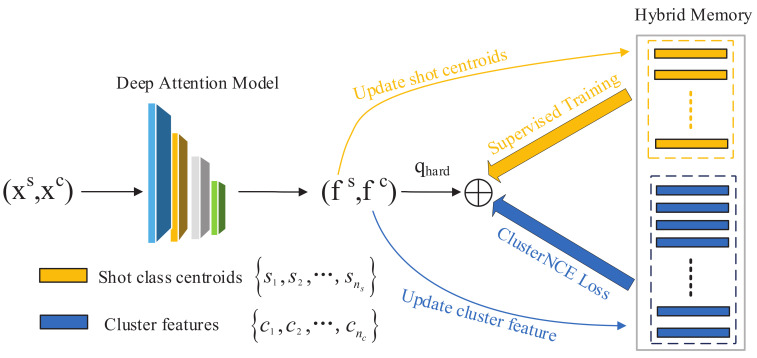
Framework of our proposed method with a hybrid memory. Firstly, the one-shot data and unlabeled data are initialized by our deep attention module. Secondly, the one-shot data class centers and unlabeled data cluster features are sent to the hybrid memory. Then, we train the one-shot data features and cluster features jointly with hard instance features. Finally, we dynamically update the features of our hybrid memory.

To make full use of one-shot data to improve the performance of person Re-ID, we propose a hybrid memory update framework (Figure 2). The key innovation of our method is that we integrate the clustering algorithm for unsupervised domains into one-shot learning. Through our hybrid memory, one-shot samples can contribute to clustering learning efficiently. In order to overcome the overfitting problem derived from the deficient quantity of one-shot data, we initialize the one-shot features at first and fine-tune their features during each epoch (Algorithm 1).
**Algorithm 1** Hybrid memory update algorithm for one-shot person Re-ID**Require:** Dataset with both one-shot labeled data $X^{s}$ and unlabeled data $X^{u}$.**Require:** Initialize the backbone network with ImageNet-pretrained ResNet-50 and layers of our relation-based attention module with kaiming initialization;**Require:** Initialize the one-shot feature centers { $s_{i}$ } through features extracted by the deep network $f_{\theta }$;**Require:** Temperature τ for Equation (6), momentum $m^{s}$ for Equation (5), momentum $m^{c}$ for Equation (4);1:**for** n in [1, num_epoches] **do**2:    Extract feature vectors for $X^{u}$ by $f_{\theta }$;3:    Cluster features of $X^{u}$ into N clusters { $c_{i}$ } with DBSCAN;4:    Initialize the clustering features via { $c_{i}$ } with Equation (3);5:    Compose features { $s_{i}$ } and { $c_{i}$ } to bulid the hybrid memory;6:    **for** n in [1, num_iterations] **do**7:        Compute the unified contrastive loss with $f_{i}^{s}$ , $f_{i}^{c}$ by Equation (6) and update $f_{\theta }$ by back-propagation;8:        Update one-shot centers { $s_{i}$ } in the hybrid memory with $f_{i}^{s}$ and momentum $m^{s}$ (Equation (5));9:        Update cluster features { $c_{i}$ } in the hybrid memory with $f_{i}^{c}$ and momentum $m^{c}$ (Equation (4));10:    **end for**11:**end for**

As depicted in Algorithm 1, our method involves three parts including memory initialization, memory update, and deep network training. Then, we discuss these three processes in detail.

#### 3.2.1. Memory Initialization

Our hybrid memory initialization process can be divided into two parts. For the one-shot data $x^{s}$, class centers { $s_{i}$ } are initialized by the deep network $f_{\theta }$. For the unlabeled data, the mean of the cluster features { $c_{1}$,…, $c_{N}$ } from DBSCAN is used to initialize the cluster feature,

(3) $$c_{i}=\frac{1}{\left|N_{i}\right|}\sum X_{i}^{u},$$

where $N_{i}$ denotes the number of instances in cluster *i*, and $X_{i}^{u}$ is the *i*-th cluster set.

#### 3.2.2. Memory Updating

The feature vectors in each batch will be updated in the hybrid memory at each iteration. For unlabeled data, we select the hardest instance feature vectors from query instances to update the corresponding cluster features. The process of feature update for a certain cluster with person identity *i* is as follows:

(4) $$c_{i}\leftarrow m^{c}\cdot c_{i}+\left(1-m^{c}\right)\cdot q_{hard}$$

where $q_{\mathrm{hard}\;}$ represents the instance with the minimum similarity to the cluster feature $c_{i}$. $m^{c}$ is the momentum hyperparameter for the unlabeled data. As for the one-shot data, class centers { $s_{i}$ } will be updated by the mean of the extracted features belonging to class *i* in the mini-batch as follows:

(5) $$s_{i}\leftarrow m^{s}s_{i}+\left(1-m^{s}\right)\cdot \frac{1}{\left|Q_{i}\right|}\sum_{f_{i}^{s}\in Q_{i}}f_{i}^{s}$$

where $Q_{i}$ denotes the feature set belonging to class *i* in the current batch. The variance $Q_{i}$ means that our method can be expanded to the few-shot situation. $m^{c}$ represents a momentum coefficient for updating few-shot class centers.

#### 3.2.3. Model Training

For a general data vector *x*, $x\in X^{s}\cup X^{u}$, we build its feature vector *f* = $f_{\theta }\left(x\right)$ and train the model uniformly as follows:

(6) $$L_{f}=-\log\frac{\exp\left((f\cdot z^{+})/\tau \right)}{\lambda \cdot \sum_{i=1}^{n^{s}}\exp\left((f\cdot s_{i})/\tau \right)+\sum_{i=1}^{n^{c}}\exp\left((f\cdot c_{i})/\tau \right)}$$

where $z^{+}$ represents the positive class prototype corresponding to *f*, and τ is a temperature hyper-parameter. $n^{s}$ and $n^{c}$ denote the number of one-shot feature centers and the number of clusters from DBSCAN, respectively. λ is a coefficient for the one-shot features. Specifically, $z^{+}=\left\{s_{i}\right\}$ is the center of the one-shot data class *i*, and $z^{+}=\left\{c_{i}\right\}$ is positive cluster feature vector to feature *f*.

**Discussion.** Compared with contrast cluster, our method extends it from an unsupervised algorithm to a one-shot framework and makes notable improvement in the person Re-ID task. At the same time, our update mode of hybrid memory overcomes the overfitting problem considering the limit of the number of one-shot data. This helps to generalize our method to other scenes where only a small number of labeled data can be obtained.

### 3.3. Relation-Based Attention Module

**Figure 3:**
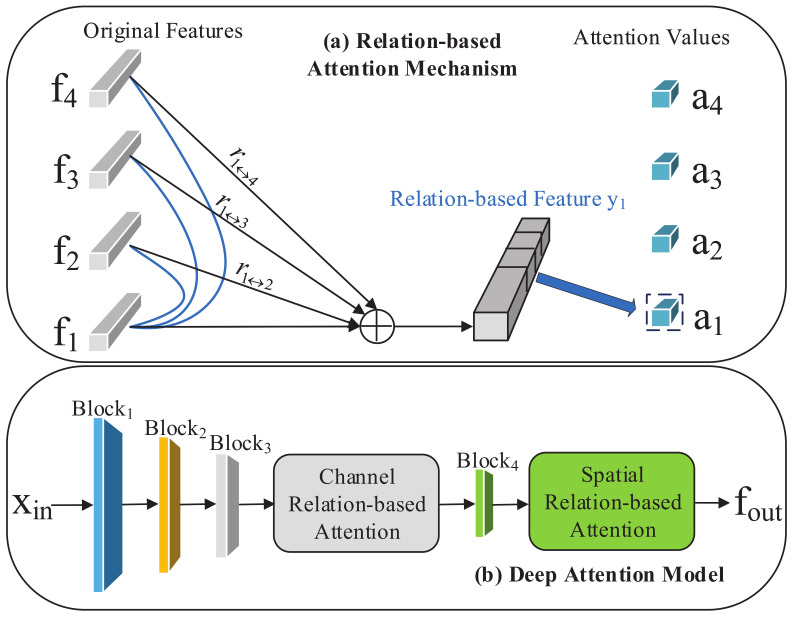
Illustration of our relation-based attention module: (**a**) an example of building the relation information with feature $f_{1}$. The feature of each sample is associated with all other samples before obtaining its group-wise attention value; (**b**) details of our deep attention module. We add our designed channel relation-based attention module and spatial relation-based attention module to block3 and block4 of ResNet50 separately.

Referred to RGA, we make full use of the original and relational information to improve the model training process. As illustrated in Figure 3a, the original feature $f_{1}$ and its relation vector $r_{1}$ are concatenated to get the relation-based feature $y_{1}$ for further training. It is worth noting that $r_{1}$ = [$r_{1,2},\cdots ,r_{1,4},r_{2,1},\cdots ,r_{4,1}$] is built by stacking the pairwise relations and $r_{i↔j}$ = [$r_{i,j},r_{j,i}$].

CBAM introduces the spatial and channel attention module into the deep network to extract important features. Then, the relation-based features need to be integrated into the attention module. The pairwise relation $r_{i,j}$ from node *i* to node *j* can be denoted as a dot-product affinity in the embedding spaces as follows:

(7) $$r_{i,j}=f_{m}\left(x_{i},x_{j}\right)=\theta_{m}{\left(x_{i}\right)}^{T}\phi_{m}\left(x_{j}\right)$$

where θ and ϕ are two embedding functions implemented by a 1 × 1 Conv layer followed by batch normalization (BN) [29] and ReLU activation. *m* means the mode for spatial attention or channel attention.

To make the relation-based feature y adapt to the spatial and channel attention mechanism, the relational feature ${\tilde{y}}_{i}$ is defined as

(8) $${\tilde{y}}_{i}=\left[{\mathrm{pool}}_{c}\left(\psi_{m}\left(x_{i}\right)\right),\varphi_{m}\left(r_{i}\right)\right]$$

where ψ and φ denote the embedding functions similar to Equation (7) for the original feature and its relational feature.

To learn the features with renewable parameters, the attention value $a_{i}$ for the $i^{th}$ node is achieved through the function as follows:

(9) $$a_{i}=\mathrm{Sigmoid}\left(W_{2}\mathrm{ReLU}\left(W_{1}{\tilde{y}}_{i}\right)\right)$$

where $W_{1}$ and $W_{2}$ are implemented by 1 × 1 convolution followed by BN.

However, considering the black-box model of neural networks and the CBAM framework, which is specially applied for supervised tasks, we need to carefully design the structure of our deep network based on the cluster features. By comparing the performance of spatial- and channel-relation-based attention solely and jointly, we design our deep attention model shown in Figure 3b, which significantly outperforms the state-of-the-art methods for the one-shot person Re-ID task.

## 4. Experiments

### 4.1. Datasets

As shown in Table 1, we evaluated our method based on three widely used datasets including Market-1501 [30], DukeMTMC-reID [31,32] and MSMT17 [33]. The latest dataset, MSMT17, has the most images and is most challenging among the above datasets.

In our experiment, both the cumulative match characteristic (CMC) curve and the mean average precision (mAP) were used to evaluate the performance among all above datasets. The CMC curve means precision of correcting with different ranking numbers, and the mAP represents the mean of the average precision (AP) for all query images.

### 4.2. Implementation Details

We added our relation-based attention module into ResNet-50 [34], following Figure 3b as the feature extractor, and initialized the backbone of the model with the parameters pretrained on ImageNet [35]. After the spatial relation-based attention module, we removed all sub-module layers and added global average pooling (GAP), followed by batch normalization layer and L2-normalization layer with 2048-dimensional features. For each epoch, DBSCAN was used to generate cluster labels.

The input images from Market-1501 and DukeMTMC-reID were resized to 256 × 128, and from MSMT17 datasets were resized to 224 × 224. During the training stage, we acquired data augmentation including random horizontal flipping, padding with 10 pixels, random cropping, and random erasing [36]. Different from our baseline, cluster contrast, which adapts 4 GPU for training and heavily depends on batch sizes, we only used 2 GTX-1080TI GPUs for training. Considering the restriction of our GPU memory, each mini-batch contained 100 images of 8 pseudo-person identities, which is about half, compared with the batch size of cluster contrast. Adam optimizer was used to train the deep model with a weight decay of 0.00005. The initial learning rate was set to 0.00035, and it was reduced to 1/10 of its previous value every 20 epoch in a total of 50 epochs. To demonstrate the effectiveness of our method, we followed the DBSCAN parameter k = 30 similar to cluster contrast and chose d as 0.4, 0.5, and 0.7 for Market-1501, DukeMTMC-reID, and MSMT17, respectively. The temperature τ in Equation (6) was set as 0.05, and the momentum parameters were fixed $m^{s}$ = $m^{c}$ = 0.2 for all datasets.

In this section, we compare our method with the state-of-the-art methods for Re-ID. Because only relatively few people study Re-ID with one example, we introduce some USL and UDA methods additionally for comparison. For a fair comparison, we reproduced the work of cluster contrast with the same hardware environment, 2 GTX-1080TI GPUs. In this case, each mini-batch contained 128 images of 8 pseudo-person identities, and other parameters remain unchanged.

### 4.3. Comparison with State-of-the-Art Methods

As shown in Table 2, our method achieves the best performance, compared with all USL methods, UDA methods, and one-shot methods. In this part, we reproduce the previous methods according to their original papers and obtain the same performance as in their papers. Compared with the USL methods, the mAP of our method surpasses the state-of-the-art USL method cluster contrast 6.7%, 4.6%, and 11.5% on Market-1501, DukeMTMC-reID, and MSMT17, respectively. Especially on MSMT17, which is the most challenging dataset, our improvement is exciting. As for the UDA methods, which can make full use of the labeled source domain dataset, Table 2 shows that our performance outperforms the state-of-the-art method SPCL up to 8.9%, 6.7%, 17.9% separately. Since we innovatively introduce the clustering algorithm into a one-shot situation, the mAP of our method surpasses the state-of-the-art one-shot method by up to 43.7% and 35.2% on Market-1501 and DukeMTMC-reID, respectively.

### 4.4. Training and Inference Cost

Through adding the relation-based attention module and additionally introducing one-shot labeled data, it is self-evident that our method requires more cost than our baseline, cluster contrast. To balance the cost and performance of our method, we conducted experiments on three datasets to compare with the state-of-the-art USL method, cluster contrast. Each result was obtained from the average of three trials.

From Table 3, we can see that our method indeed incurs additional costs during the training stage. Importantly, our model has a similar cost during the inference stage, compared with the baseline. This is because the deep network is only used for feature extraction, and then the traditional ranking algorithm for person Re-ID is taken to query the corresponding identity. In this case, the inference time depends on the size of the query dataset. Considering that the task of person Re-ID task is not a real-time task, our method has broad prospects for promotion in the field of social security.

### 4.5. Experiment Initialization and Implementation

In this section, we study the influence of different parameter values on our method and show all the parameters for initialization on three datasets in the end. Our experiment is based on the state-of-the-art USL method, cluster contrast (training parameters: https://github.com/alibaba/cluster-contrast-reid, accessed on 30 April 2021). Both the performance of our method and cluster contrast are positively correlated with the size of mini-batch. Considering the device environment of 2 GTX-1080TI GPUs, we set the batch size of all experiments to 100. Then, we studied the following hyperparameters for the optimal initialization. Other hyperparameters in our method remained unchanged.

#### 4.5.1. Influence of Training Iterations

**Figure 4:**
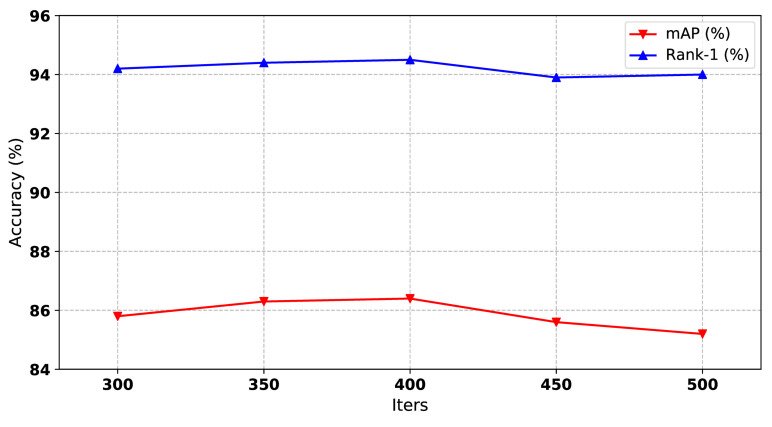
The impact of training iterations of our method on Market1501 dataset. Both mAP and Rank-1 are used to evaluate the performance. The x-axis represents the number of training iterations.

In order to train the model to converge and avoid overfitting, we tested the performance of our method in different iterations. From Figure 4, we can see that the iteration of 400 is optimal. More or fewer iterations will degrade the performance.

#### 4.5.2. Influence of Momentum Values

**Figure 5:**
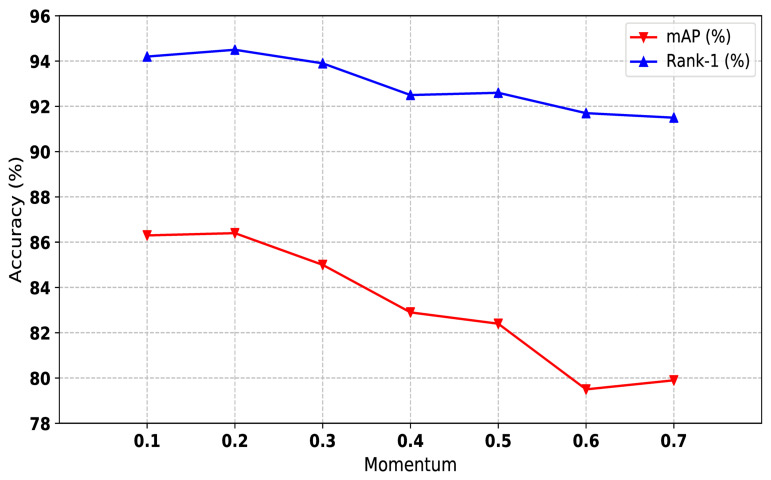
The impact of momentum values of our method on Market1501 dataset. Both mAP and Rank-1 are used to evaluate the performance. The x-axis represents momentum values.

We used the momentum update strategy to fine-tune cluster features in our hybrid memory. As shown in Equations (4) and (5), the momentum value *m* controls the update speed of cluster memory. The larger the value of *m* is, the slower the hybrid memory updates. We conducted experiments on Market-1501 to explore the influence of different *m* values. As shown in Figure 5, smaller *m* (less than 0.3) performs better than larger *m* (greater than 0.4). It is worth noting that the consistent value of $m^{s}$ and $m^{c}$ obtains the best performance. Considering that the momentum value is unrelated to the dataset, we set $m^{s}$ and $m^{c}$ to 0.2 on all datasets.

#### 4.5.3. Influence of the Feature Learning Coefficient of Labeled Data

**Figure 6:**
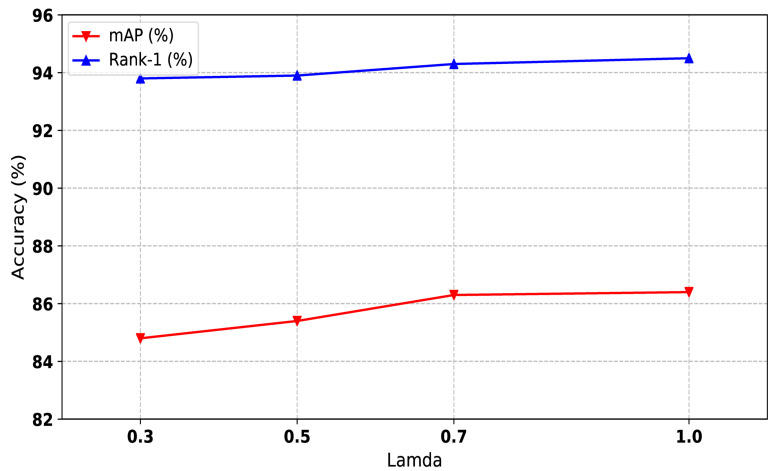
The impact of the feature learning coefficient of labeled data of our method on Market1501 dataset. Both mAP and Rank-1 are used to evaluate the performance. The x-axis represents the joint training weights.

We trained the one-shot labeled data and unlabeled data uniformly. From Equation (6), we can see that λ controls the weight of loss computing. To make sure the influence of the feature learning coefficient of one-shot data, we conducted experiments among different values of λ, and the result is shown in Figure 6. λ is also a hyperparameter that is indepent of the type of dataset; thus, we set λ to 1 for all experiments.

#### 4.5.4. Influence of Clustering Hyperparameters

**Figure 7:**
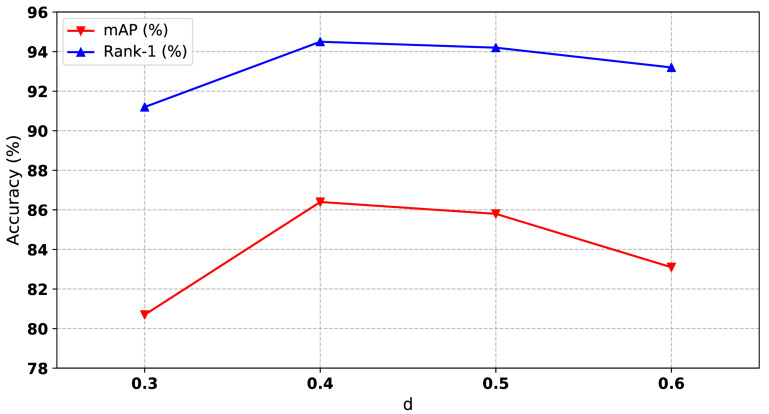
Performances of our method with different of *d*, where *d* represents the maximum distance between two samples in DBSCAN algorithm.

The maximum distance *d* between two samples is a hyperparameter of the DBSCAN algorithm, which will affect the final number of clusters. If the *d* value is chosen smaller, then a larger part of the data will be considered as outliers. If it is chosen larger then the clusters will merge and a majority of the data points will be in the same clusters. Importantly, it contributes to reducing the negative effect of the hard instance on our relation-based attention module. From Figure 7, we can see that the value of *d* has a relatively large impact on the results, and the values of *d* for the optimal results are different under different datasets. The values of *d* on different datasets will be listed at the end of this section.

#### 4.5.5. Influence of Instance Number

**Figure 8:**
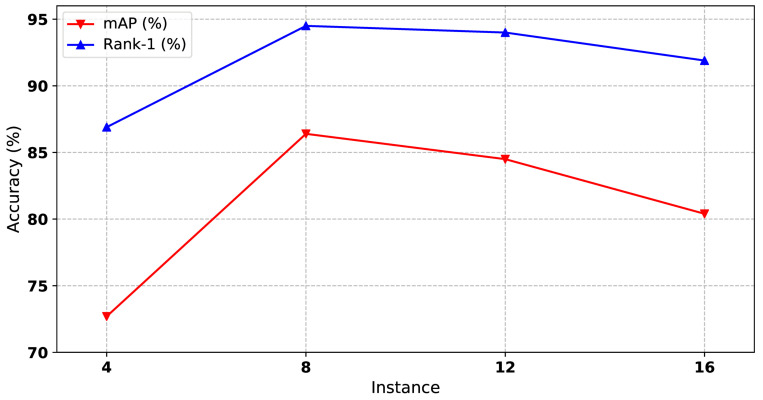
The impact of instance number to our method on Market1501 dataset. Both mAP and Rank-1 are used to evaluate the performance. The x-axis represents the number of instances for each mini-batch.

Considering that our clustering algorithm depends on the size of the mini-batch, the performance of our method increases as the batch size increases. However, the mini-batch size is limited due to the GPU memory. In our experiments, we set the batch size to 100 as the upper bound. To deal with the limitation of the GPU memory, we fix the batch size to a constant number, and the number of instances in each mini-batch varies. As shown in Figure 8, the optimal number of instances is 8, corresponding to our mini-batch size, 100.

Simply put, some parameters including momentum values, λ, and the number of instances are universal and consistent across different datasets. λ and *d* need to be set specifically for different tasks. Our initialization parameters are shown in Table 4. It is worth noting that the hard instance update mode for MSMT17 dataset causes performance degradation, which can be due to the characteristics of the challenging MSMT17 dataset. As a result, we updated the cluster feature with all the instance features in each mini-batch and set the training iteration to 400.

### 4.6. Ablation Studies

In this section, we study the effectiveness of various components in our method. We define the unsupervised method cluster contrast as the **baseline** method.

#### 4.6.1. Ablation Study on the Framework Components

To investigate the impact of each component in our method, we disabled some components of our method, and the ablation study was conducted on the Market1501 dataset. Firstly, we added our relation-based attention module into the baseline and called it **Attention** for short. Then, we only introduced our hybrid memory for one-shot data without modifying any structure of the backbone, ResNet-50. Finally, we integrated the above two components to train the model for person Re-ID.

Table 5 shows the results of our experiments. Compared with the baseline, the method with our relation-based attention module improves mAP by 3.6 on the Market1501, which proves the contribution of our attention structure design. Compared with the baseline, our hybrid memory for one-shot data improves the performance of mAP, reaching 4.1 on Market1501. Finally, our method of combining the above components achieves further success.

#### 4.6.2. Ablation Study on Attention Modules

**Figure 9:**
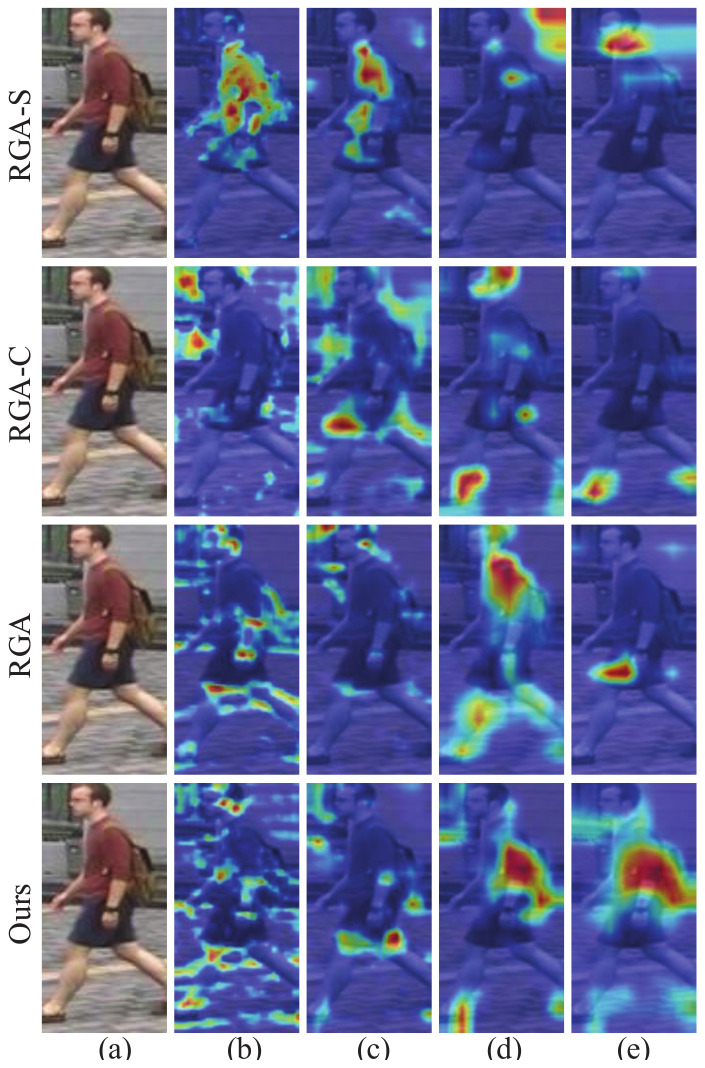
Grad-CAM visualization for attention modules from different blocks: (**a**–**e**) correspond to the original image and the outputs from the attention module after block1, block2, block3, block4 of ResNet-50. For our model, (**a**,**b**) represent the outputs from block1 and block2, respectively.

Although RGA makes progress in the supervised person Re-ID task, considering that our clustering-based algorithm differs from the supervised method, we naturally considered carefully designing the structure of the attention module to generalize the relation-based module. In this part, we compare our methods with RGA, spatial relation-aware of RGA (RGA-S) and channel relation-aware of RGA (RGA-C). In Table 6, we can see that our relation-based attention module wins the optimal performance. It is surprising that RGA-S and RGA-C solely cause various performance degradation, while their combination makes little progress. This inspired us to study the output features from different network modules. Then, we used the Grad-CAM [41] tool, which can identify the regions that the network considers important to verify our idea. Figure 9 shows examples from different blocks. It is evident that our attention modules after block3 and block4 (Figure 3b) capture the more accurate and wider range of person features for training. We also find that spatial and channel mechanisms pay attention to different features and play different roles after various blocks. Then, the reason for the success of our attention module is understandable.

#### 4.6.3. Ablation Study on Memory Update Mode

SPCL builds a memory that combines the source domain dataset and the target domain dataset and trains it with momentum update without modifying the structure of memory. However, cluster contrast reconstructs the memory to train the cluster at the beginning of each epoch. Therefore, the study of introducing the one-shot data into our designed hybrid memory seems important. In this section, we first trained the deep network with the memory of SPCL and the memory of cluster contrast updating asynchronously. This memory update mode uses two different memories and is short as split memory. Furthermore, we also examined a memory update mode that regards one-shot features as cluster features and is reconstructed synchronously with the cluster features at each epoch. This mode updates with a unified memory and is abbreviated synchronous memory. As is shown in Table 7, compared with split memory and synchronous memory, our hybrid memory achieves 1.5 and 2.2 performance improvement, respectively. The progress can be attributed to our unified memory design and special one-shot data update design, which overcomes the problem of overfitting.

## 5. Discussion

In this section, further analysis of our proposed method is discussed. On the one hand, we are the first to introduce the clustering algorithm to one-shot person Re-ID. Through our hybrid memory, which dynamically stores and updates the labeled features and cluster vectors, the performance of Re-ID improves significantly, compared with both USL and UDA methods. Our especially designed update mode for the one-shot data plays an important role in fine-tuning the unsupervised clustering features and helps improve performance. For a real-world situation where only unlabeled data can be obtained, annotating small numbers of data within an acceptable cost will make significant progress with our methods. From the above points of view, our approach has a great application prospect. However, considering that we only used the limited labeled data to fine-tune the clustering learning, the improvement of performance may be limited. Then, how to make full use of the labeled data to contribute to the pseudo-label annotating still has great potential for development. On the other hand, our designed relation-based deep attention module pays attention to the key information of the identity and suppresses the noise from the environment. However, the global information involved and the additional modules added to ResNet-50 increase the cost of calculation. In this situation, the hardware determines the upper limit of task performance. As a result, how to improve the performance of person Re-ID with relatively low cost may be a promising direction of development.

## 6. Conclusions

In this paper, we propose a relation-based attention module with hybrid memory, which extracts important features for training and trains the one-shot and unlabeled data jointly. It has three contributions to the one-shot person Re-ID task. Firstly, the attention module drives the deep network to strengthen the characteristics of each identity and suppress irrelevant areas. Secondly, we are the first to introduce the clustering algorithm to the one-shot person Re-ID, and the clustering evaluation criteria significantly improves the performance of pseudo-label annotation. Finally, the hybrid memory with a specially designed update mechanism overcomes the problem of overfitting caused by one-shot data and makes full use of information of one-shot images to improve the performance of Re-ID. Experiments show that our method for one-shot person Re-ID outperforms the state-of-the-art unsupervised and one-shot methods. Furthermore, beyond the person Re-ID, our method has the potential to be used for other tasks in which only a small number of labeled data can be obtained for the unlabeled dataset.

## Figures and Tables

**Table 1:** Statistics of the datasets used for training and evaluation.

**Dataset**	**Train IDs**	**Train Images**	**Test IDs**	**Query Images**	**Cameras**	**Total Images**
Market-1501	751	12,936	750	3368	6	32,217
DukeMTMC-reID	702	16,522	702	2228	8	36,441
MSMT17	1041	32,621	3060	11,659	15	126,441

**Table 2:** Comparison with state-of-the-art methods on the person Re-ID, including one-shot methods, USL methods, and UDA methods. **None** represents the USL method. **One-shot** means the one-shot method. The other value represents the source-domain dataset in UDA method.

**Methods**	**Market-1501**				
	**Source**	**mAP**	**Top-1**	**Top-5**	**Top-10**
SSL [37]	None	37.8	71.7	83.8	87.4
MMCL [9]	None	45.5	80.3	89.4	92.3
MMCL [9]	Duke	60.4	84.4	92.8	95.0
HCT [38]	None	56.4	80.0	91.6	95.2
CycAs [39]	None	64.8	84.8	-	-
AD-Cluster++ [25]	Duke	68.3	86.7	94.4	96.5
UGA [40]	None	70.3	87.2	-	-
SPCL [7]	None	73.1	88.1	95.1	97.0
SPCL [7]	MSMT17	77.5	89.7	96.1	97.6
EUG [14]	One-shot	26.2	55.8	72.3	78.4
PPLS [15]	One-shot	42.7	74.6	86.3	90.1
Cluster Contrast [10]	None	79.7	91.0	95.6	96.9
**Ours**	One-shot	**86.4**	**94.8**	**97.9**	**98.6**
**Methods**	**DukeMTMC-reID**				
	**Source**	**mAP**	**Top-1**	**Top-5**	**Top-10**
SSL [37]	None	28.6	52.5	63.5	68.9
MMCL [9]	None	51.4	72.4	82.9	85.0
AD-Cluster++ [25]	Market	54.1	72.6	82.5	85.5
MMCL [9]	Market	51.4	72.4	82.9	85.0
HCT [38]	None	50.7	69.6	83.4	87.4
UGA [40]	None	53.3	75.0	-	-
CycAs [39]	None	60.1	77.9	-	-
SPCL [7]	None	65.3	81.2	90.3	92.2
SPCL [7]	Market	68.8	82.9	90.1	92.5
EUG [14]	One-shot	28.5	48.8	63.4	68.4
PPLS [15]	One-shot	40.3	64.6	75.2	79.1
Cluster Contrast [10]	None	70.9	82.9	90.8	93.8
**Ours**	One-shot	**75.5**	**86.4**	**92.7**	**94.9**
**Methods**	**MSMT17**				
	**Source**	**mAP**	**Top-1**	**Top-5**	**Top-10**
MMCL [9]	None	11.2	35.4	44.8	49.8
SPCL [7]	None	19.1	42.3	55.6	61.2
UGA [40]	None	21.7	49.5	-	-
CycAs [39]	None	26.7	50.1	-	-
SPCL [7]	Market	26.8	53.7	65.0	69.8
Cluster Contrast [10]	None	33.2	63.3	73.5	77.6
**Ours**	One-shot	**44.7**	**73.4**	**83.2**	**86.4**

**Table 3:** Comparison of training and inference costs.

**Methods**	**Dataset**	**Training Time**	**Inference Time**
Cluster Contrast	Market-1501	2 h 37 min	62 s
	DukeMTMC-reID	3 h 4 min	63 s
	MSMT17	6 h	9 min 54 s
Ours	Market-1501	6 h 18 min	62 s
	DukeMTMC-reID	7 h 2 min	65 s
	MSMT17	9 h 30 min	10 min 8 s

**Table 4:** Parameters for our method on different datasets.

**Dataset**	**Iters**	**Momentum**	λ	***d***	**Instance**
Market-1501	400	0.2	1	0.4	8
DukeMTMC-reID	450	0.2	1	0.5	8
MSMT17	400	0.2	1	0.7	8

**Table 5:** Ablation study of our proposed framework on individual components.

**Methods**	**Market1501**			
	**mAP**	**Rank-1**	**Rank-5**	**Rank-10**
baseline	79.7	91.0	95.6	96.9
baseline + Attention	83.3	92.5	96.9	97.8
baseline + One-shot	83.8	92.7	97.3	98.4
**baseline + attention + One-shot**	**86.4**	**94.8**	**97.9**	**98.6**

**Table 6:** Performance comparisons of our attention model with the baseline and other attention module designs. RGA-S and RGA-C, respectively, represent that only the spatial attention mechanism and the channel attention mechanism are used for relation-based features.

**Methods**	**Market1501**			
	**mAP**	**Rank-1**	**Rank-5**	**Rank-10**
baseline	79.7	91.0	95.6	96.9
baseline + RGA-S	75.6	88.6	94.2	95.7
baseline + RGA-C	68.6	84.0	91.2	93.8
baseline + RGA	80.9	91.5	96.3	97.4
**baseline + Attention**	**83.3**	**92.5**	**96.9**	**97.8**

**Table 7:** Comparisons between our hybrid memory update mechanism and other memory update mechanism designs.

**Methods**	**Market1501**			
	**mAP**	**Rank-1**	**Rank-5**	**Rank-10**
baseline	79.7	91.0	95.6	96.9
baseline + Split Memory	82.3	92.4	97.3	98.0
baseline + Synchronous Memory	81.6	92.1	96.8	97.9
**baseline + Hybrid Memory**	**83.8**	**92.7**	**97.3**	**98.4**

## Data Availability

The datasets involved in this paper are all public datasets.
